
JOURNAL INFORMATION
==============================
NLM Title Abbreviation: Innov Aging
Journal ID: Innov Aging
NLM Journal ID: 101703706
Journal ID: innovateage

Innovation in Aging

EISSN: 2399-5300
Publisher: Oxford University Press

ARTICLE INFORMATION
==============================
PMCID: PMC10735656
DOI: 10.1093/geroni/igad104.0330
Article ID: igad104.0330
Article version: 1
Subjects: Abstracts, Session 1325 (Biological Sciences Invited Symposium), AcademicSubjects/SOC02600

EVALUATING CIRCULATING GEROSCIENCE BIOMARKERS

Basisty Nathan
Publication date: 2023 Dec
Electronic publication date: 2023 Dec 21
Volume: 7
Issue: Suppl 1
Issue title: Program Abstracts from The GSA 2023 Annual Scientific Meeting, “Building Bridges > Catalyzing Research > Empowering All Ages”
First page: 102
Last page: 102
Copyright: © The Author(s) 2023. Published by Oxford University Press on behalf of The Gerontological Society of America.
Copyright year: 2023
License: This is an Open Access article distributed under the terms of the Creative Commons Attribution License (https://creativecommons.org/licenses/by/4.0/), which permits unrestricted reuse, distribution, and reproduction in any medium, provided the original work is properly cited.
License URL: https://creativecommons.org/licenses/by/4.0/

Page count: 1

==============================

Abstract citation ID: igad104.0330

**EVALUATING CIRCULATING GEROSCIENCE BIOMARKERS**

Nathan Basisty, *National Institute on Aging, Baltimore, Maryland, United States*
